# A Scoping Review of Vitamins Detection Using Electrochemically Polymerised, Molecularly Imprinted Polymers

**DOI:** 10.3390/polym17101415

**Published:** 2025-05-21

**Authors:** Mohd Azerulazree Jamilan, Balqis Kamarudin, Zainiharyati Mohd Zain, Kavirajaa Pandian Sambasevam, Faizatul Shimal Mehamod, Mohd Fairulnizal Md Noh

**Affiliations:** 1 Nutrition, Metabolism & Cardiovascular Research Centre, Institute for Medical Research, National Institute of Health, Ministry of Health Malaysia, No. 1, Jalan Setia Murni U13/52, Setia Alam, Shah Alam 40170, Selangor, Malaysia; balqis.kamarudin@moh.gov.my (B.K.); fairulnizal@moh.gov.my (M.F.M.N.); 2 Electrochemical Material and Sensors (EMaS) Research Group, Faculty of Applied Sciences, Universiti Teknologi MARA, Shah Alam 40450, Selangor, Malaysia; kavirajaa@uitm.edu.my; 3 Advanced Materials for Environmental Remediation (AMER), Faculty of Applied Sciences, Universiti Teknologi MARA, Cawangan Negeri Sembilan, Kampus Kuala Pilah, Kuala Pilah 72000, Negeri Sembilan, Malaysia; 4 Advanced Nano Materials (ANoMA) Research Group, Faculty of Science and Marine Environment, Universiti Malaysia Terengganu, Kuala Nerus 21030, Terengganu, Malaysia; fshimal@umt.edu.my

**Keywords:** in situ electro-polymerisation, eMIP, water-soluble vitamin, fat-soluble vitamin, electrochemical detection

## Abstract

Vitamins are crucial micro-nutrients for overall well-being, making continuous monitoring essential. There are demands to provide an alternative detection, especially using a portable detection or a point-of-care-testing (POCT) device. One promising approach is employing an in situ electro-polymerised MIP (eMIP), which offers a straightforward polymerisation technique on screen-printed electrodes (SPEs). Here, we report a review based on three databases (PubMed, Scopus, and Web of Science) from 2014 to 2024 using medical subject heading (MeSH) terms “electrochemical polymerisation” OR “electropolymerisation” crossed with the terms “molecularly imprinted polymer” AND “vitamin A” OR “vitamin D” OR “vitamin E” OR “vitamin K” OR “fat soluble vitamin” OR “vitamin B” OR “vitamin C” OR “water soluble vitamin”. The resulting 12 articles covered the detection of vitamins in ascorbic acid, riboflavin, cholecalciferol, calcifediol, and menadione using monomers of catechol (CAT), 3,4-ethylenedioxythiophene (EDOT), o-aminophenol (oAP), o-phenylenediamine (oPD), pyrrole, p-aminophenol (pAP), p-phenylenediamine (pPD), or resorcinol (RES), using common bare electrodes including graphite rod electrode (GRE), glassy carbon electrode (GCE), gold electrode (GE), and screen-printed carbon electrode (SPCE). The most common electrochemical detections were differential pulse voltammetry (DPV) and linear sweep voltammetry (LSV). The imprinting factor (IF) of the eMIP-modified electrodes were from 1.6 to 21.0, whereas the cross-reactivity was from 0.0% to 29.9%. Several types of food and biological samples were tested, such as supplement tablets, poultry and pharmaceutical drugs, soft drinks, beverages, milk, infant formula, human and calf serum, and human plasma. However, more discoveries and development of detection methods needs to be performed, especially for the vitamins that have not been studied yet. This will allow the improvement in the application of eMIPs on portable-based detection and POCT devices.

## 1. Introduction

Vitamins are essential micro-nutrients that the human body requires to maintain normal growth and activity of the body, as well as overall health [1]. Vitamins are obtained naturally from plant and animal foods [2], although some of them can be produced naturally from our bodies [3,4]. Vitamins are generally divided into two categories: water-soluble vitamins (WSVs) including vitamin B_1_ (thiamine), vitamin B_2_ (riboflavin), vitamin B_3_ (niacin), vitamin B_5_ (pantothenic acid), vitamin B_6_ (pyridoxine), vitamin B_7_ (biotin), vitamin B_9_ (folic acid), vitamin B_12_ (cobalamin), and vitamin C (ascorbic acid); and fat-soluble vitamins (FSVs) including vitamin A (retinol, β-carotene), vitamin D_2_ (ergocalciferol), vitamin D_3_ (cholecalciferol), vitamin E (α-tocopherol), vitamin K_1_ (phylloquinone), vitamin K_2_ (menaquinone), and vitamin K_3_ (menadione). WSVs dissolve easily in water and are not stored in the body, which makes regular dietary intake crucial, whereas in contrast, FSVs dissolve in fats and oils, allowing them to be stored in the liver before they are absorbed in the bloodstream [4]. However, most vitamins cannot be produced naturally in the body, and the ones that are naturally synthesised produce insufficient amounts, necessitating the need for regular dietary intake to meet the recommended levels.

Each vitamin is important and has its role in the human body. Avitaminosis, or vitamin deficiency, can lead to many serious diseases. For example, vitamin B1 deficiency can cause beriberi (cardiomyopathy with oedema and lactic acidosis), Wernicke–Korsakoff syndromes, or even several neurodegenerative diseases, such as Alzheimer’s disease, Parkinson’s disease, and Huntington’s disease [5]. A deficiency in vitamin C can lead to scurvy, which causes symptoms including muscle weakness, swollen and bleeding gums, loss of teeth, petechial haemorrhaging, spontaneous ecchymoses, anaemia, impaired wound healing, hyperkeratosis, weakness, myalgia, arthralgia, and weight loss [6]. Vitamin D, which regulates calcium balance and supports bone mineralisation, can cause rickets in infants or children and osteomalacia in adults if deficient [3]. These consequences emphasise the importance of maintaining optimal vitamin levels to prevent deficiency-related diseases.

The summary of vitamins with their respective active metabolites form, dietary sources, and roles in the human body are simplified in Table 1. For example, WSVs are commonly found in fruits, vegetables, whole grains, and fortified cereals, whereas FSVs are typically present in high-fat foods such as dairy products, fish, eggs, liver, and plant oils.

The detection of most analytes and other metabolites, including vitamins, biogenic amines, amino acids, and even proteins or peptides, commonly relied on laboratory-based instrumentation [9,10] such as High-Performance Liquid Chromatography (HPLC), which was typically coupled with several detectors, including ultraviolet (UV) or mass spectrometry (MS) [11,12]. This was because HPLC was considered a reference standard due to its high reliability, exceptional sensitivity, and selectivity, although the laboratory-based instrumentation incurred significant acquisition and maintenance costs. Furthermore, operating these instruments required specialised skills, encompassing method development, sample preparation, and data analysis, which often rendered the technique less practical for routine or on-site testing [13].

Consequently, there was a growing demand for alternative, portable devices that could complement or even replace traditional laboratory-based systems. These devices included portable electrochemical sensors, nonlinear optical (NLO) sensors, and point-of-care testing (POCT) platforms [14,15,16]. They offered numerous advantages such as simplicity of operation, high sensitivity, and lower costs. These features enabled real-time monitoring of various analytes, including vitamins, across different sample matrices, such as food products and biological fluids. These benefits alleviated the complexities and limitations associated with traditional analytical methods. In this context, molecularly imprinted polymer (MIP)-based electrochemical sensors emerged as highly promising candidates. MIP-based sensors provided excellent selectivity by mimicking the functions of biological recognition elements while remaining cost-effective, robust, and highly adaptable for integration into portable systems and POCT devices.

**Figure 1:**
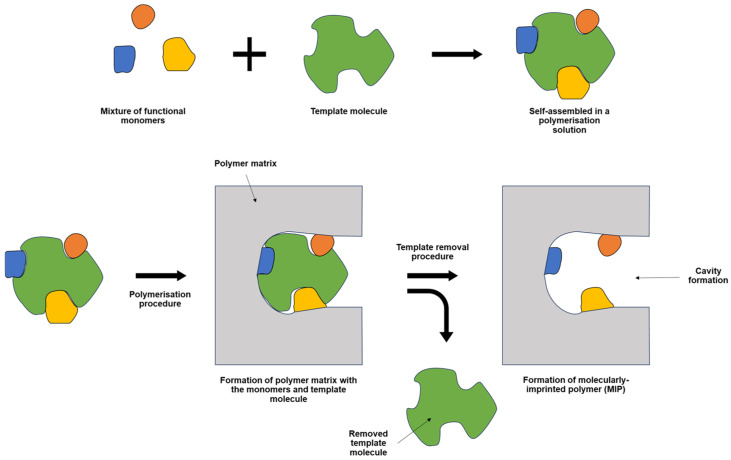
The general overview for the synthesis of MIP.

An MIP is a polymer specifically designed to match the shape of a template molecule. While the target compound itself is commonly used as the template, a structurally similar compound can also serve this purpose [17]. The general overview of MIP formation is illustrated in Figure 1 below. The polymerisation solution of MIP can be prepared by mixing several key ingredients, including the monomer(s), the template, a crosslinker (if required), and an electrolyte [18]. In the polymerisation solution, one or several monomers can be used to form MIP, depending on the desired MIP design. The easiest monomer(s) to be used was the self-polymerising monomer, which is a monomer that can interlink with the same monomer without the presence of any crosslinker [19]. This can reduce the use of too many parameters for optimisation of the MIP procedure, thereby shortening the overall optimisation steps. The template removal, one of the critical steps in the formation of effective MIPs, was enacted to leach out any template residual on the MIP structure, leaving a space or cavity, specific to the target compound. This contributed to the high specificity of the MIP [20]. Also, the introduction of the electrolyte in the polymerisation solution was required to facilitate ion movement to the working electrode surface [21]. Any electrolyte can be used depending on its solubility on the prepared polymerisation solution composition.

On the other hand, an electrochemical detection platform is always a good alternative for detection, as the technique can be applied using a miniaturised and portable design. The detection of an electrochemical sensor deals with the study of electron transfer between species involving redox reactions, where losses of electrons (oxidation) and gains of electrons (reduction) result in the oxidation states of one species [22,23]. These changes can be detected through the observed current signal produced by the sensor device, which acts as an electrolytic cell. There are several techniques to observe the current signal for sample detection, including cyclic voltammetry (CV), linear sweep voltammetry (LSV), differential pulse voltammetry (DPV), and square-wave voltammetry (SWV) [22]. Typically, DPV and SWV gained much more attention for analyte detection at trace levels due to their significant improvement in signal through background noise reduction, which significantly enhance the signal-to-noise ratios, thereby indirectly lowering the detection limit much further and improving detection sensitivity [24,25]. Nevertheless, depending on the application of the study, these techniques are utilised to obtain an improved and optimum detection signal for quantification [26,27]. This capability makes them invaluable tools in modern analytical chemistry.

Thus, the combination of electrochemical detection using electrodes that were modified with MIP has recently gained popularity due to the high specificity results from MIP [18]. Moreover, the electrochemical polymerisation (electro-polymerisation) of MIP, denoted as eMIP, is also possible to be designed using an electrochemical technique, making the combination of eMIP and electrochemical detection a good option to explore for an alternative, affordable, reliable, and portable detection of vitamin with simple electro-polymerisation procedures. However, the technique also relies on the type of target compound to be detected. Not every target compound can be detected directly by electrochemical detection due to its electrochemically active nature.

For instance, certain vitamins, especially their metabolites such as calcifediol [28] and cobalamin [29], are unable to produce a measurable current signal due to their electrochemical inactivity. This inactivity is caused by their non-polar structures and the lack of strongly redox-active functional groups, such as phenolic hydroxyls [30], or quinone-like structures [31], which limit their ability to undergo oxidation or reduction within the typical potential range of most electrodes. Additionally, these metabolites exhibit high electron transfer overpotentials and do not have easily ionisable groups, which makes direct electrochemical detection challenging without modification or the use of mediators [32,33].

Nevertheless, this limitation can be addressed by monitoring the signal of a mediator such as a redox indicator on an eMIP-modified electrode before and after incubation with non-electrochemically active vitamins. The alteration in the redox indicator’s response demonstrates the binding interaction between the target metabolites and the MIP, thus facilitating indirect electrochemical detection. Therefore, the aim of this review was to evaluate the most applicable technique for performing electro-polymerisation on a variety of electrode materials, including carbon-based, metal, and composite electrodes, and to discover the need for developing innovative electrochemical-sensing strategies to detect vitamins in different samples.

## 2. Results

In the initial stage of screening, the bibliometric search results found 46 publications where 28 out of the 46 articles were excluded based on inclusion and exclusion criteria. Articles that have met the requirements, including articles with full text, reported in English, and peer-reviewed, were included to ensure the selection of high-quality and relevant research. The exclusion of articles were letters to the editor, books or book chapters, review papers, or duplicates, as these did not contribute original research data or were redundant. The search strategy also applied a filter for articles that were published from 10 years ago up until the present day (2014–present). This was to ensure our review reflects the current and latest knowledge.

**Figure 2:**
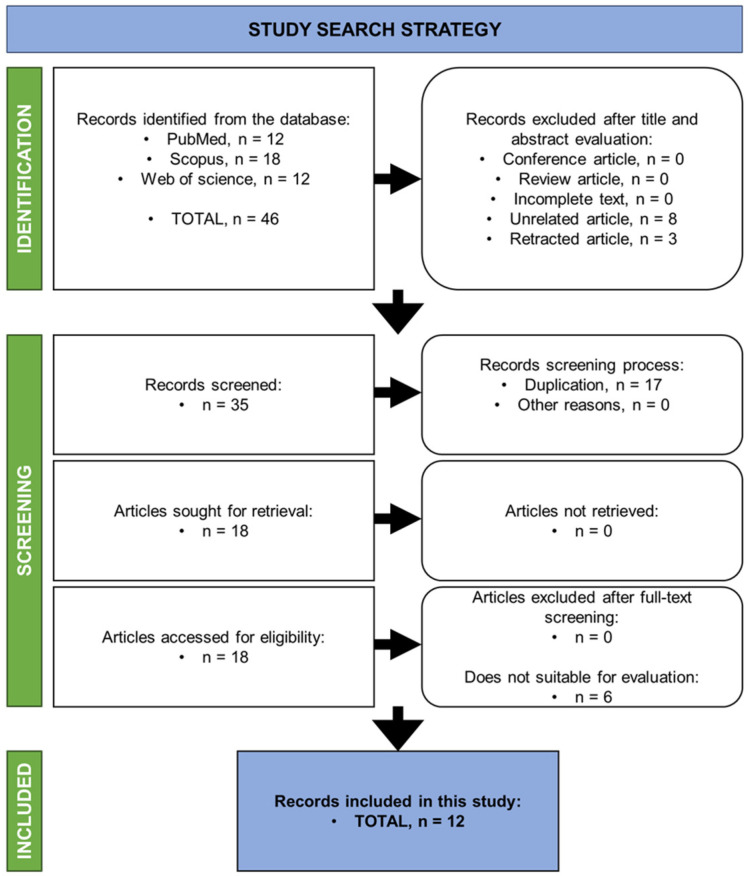
The summarised search strategy of the study.

Based on the article searches from the databases, and following the rules set within the inclusion and exclusion criteria, 18 articles were found. Only 12 full-text publications were deemed relevant after full screening for each article was performed (Figure 2).

Furthermore, a summary of each article was tabulated in Table 2 below to better understand the whole context of the articles. The related information was categorised according to the aim, methodology, findings, and conclusion for a more thorough analysis of the articles. It is important to note that the Results and Discussion sections specify the chemical names of the vitamins to clearly identify the exact target compounds used in the study.

## 3. Discussion

The electro-polymerisation technique is one of the simplest approaches for polymerisation, which can also be utilised to form an MIP by introducing a template into the polymerisation solution [18]. The spontaneous formation of MIP on the working electrode surface after the electro-polymerisation procedures were performed led to an in situ formation of eMIP. Studies have also reported the polymerisation of MIP conducted elsewhere, typically through chemical reaction, where the formed MIP was collected and modified on the sensing electrode [45,46]. This process requires additional preparation steps.

The polymerisation of the monomer(s) can be initiated using an electrochemical technique through oxidation or reduction [21,47]. This occurs when a specific potential was applied to the polymerisation solution. A bare conventional electrode is then immersed in the polymerisation solution, after which the electrochemical technique is applied to initiate polymerisation. In the case of screen-printed electrodes (SPEs), they can be immersed in the polymerisation solution, or a portion of the polymerisation can be placed on the SPEs’ surface prior to electro-polymerisation.

To assess the specificity of the formed MIP, the same polymerisation solution is typically prepared and modified on the same bare electrode but without the template, which is known as a non-imprinted polymer (NIP). The specificity of the eMIP-modified electrode can be compared by calculating the MIP and NIP ratio, often referred to as the imprinting factor (IF). An IF of greater than one (IF > 1) indicates that the MIP was specific [48], although a higher IF value is preferable. However, an excessively high IF could indicate a higher binding affinity of the target compound towards the MIP, which might present challenges for template or target compound removal [49]. Residual target compound, due to ineffective template removal procedures, can lead to poor sensitivity of the eMIP-modified electrode [50].

In this review, the reported studies were compared by several criteria, including the MIP preparation technique, the detection of target compound, the concentration range of the detection, the detection technique, the methods of template removal, the type of real sample used, the imprinting factor (IF), and the cross-reactivity study.

The IF is usually determined by the formula shown in Equation (1):

(1) $$IF=\frac{{MIP}_{target}}{{NIP}_{target}}$$

where ${MIP}_{target}$ is the signal produced by the target compound using the MIP-modified electrode, and ${NIP}_{target}$ is the signal produced by the target compound using the NIP-modified electrode. The IF value represents a measure of the interaction affinity of a target compound towards its respective MIP in the imprinted cavity [51]. The supposedly non-interacted target compound with the NIP gives a lower signal, leading to a higher IF value. Thus, a higher IF value indicates good specificity of the synthesised MIP. However, a too-high IF value can also mean that the affinity of the target compound with the MIP is too strong, posing a challenge to removing the target compound from the MIP.

On the other hand, the cross-reactivity is calculated based on the formula given in Equation (2) [52]:

(2) $$Cross-reactivity=\frac{{MIP}_{interfering\;compound}}{{MIP}_{target}}\times 100\%$$

where the ${MIP}_{interfering\;compound}$ is the signal produced by the interfering compound using the MIP-modified electrode and ${MIP}_{target}$ is the signal produced by the target compound using the MIP-modified electrode. The cross-reactivity study was only discussed if the interference study was reported. The cross-reactivity study in MIP synthesis is a measure of non-selective interaction of non-target compounds with the imprinted cavity of the synthesised MIP. A higher percentage of the cross-reactivity value indicates that the MIP is becoming less selective towards the target compound. Typically, non-selectivity occurs when the chemical structure of the non-target compound contains a similar major backbone structure to that of the target compound, mimicking the interaction with the MIP cavity and leading to a false positive signal [52,53]. This contributes to a higher cross-reactivity percentage.

Based on the final results of scoping, there were limited studies reported regarding the detection of vitamins using the electro-polymerisation technique, in which the most common electrode used was the conventional type of electrodes such as graphite rod electrode (GRE), GCE, or even gold electrode (GE). Only very few studies utilised the use of SPEs.

For instance, the determination of ascorbic acid was successful by using the MIP of polypyrrole (PPY) on an electrospun-modified cellulose acetate nanofibre membrane (CA)/multi-walled carbon nanotubes (MWCNTs)/polyvinylpyrrolidone (PVP) on bare GCE (PPY/CA/MWCNTs/PVP/GCE), which was electro-polymerised using CV (−0.6 V to 0.8 V, 7 cycles, 100 mV/s) [34]. The polymerisation solution contains pyrrole and ascorbic acid with a molar ratio of 2.5:1 in lithium perchlorate (LiClO_4_, 0.1 M). The removal of the template was performed by immersing the eMIP-modified electrode in phosphate buffer (PB) (0.05 M, pH 8.5) solution for 15 min. The direct detection (visualised E vs. silver/silver chloride, Ag/AgCl at 0.0 V) of ascorbic acid was performed using DPV (−0.2 V to 0.4 V) after 5 min of immersion of the eMIP-modified electrode in the standard solution containing PB (0.05 M, pH 8.5). An extensive range of linear calibration was observed for ascorbic acid concentrations ranging from 20 µM to 1000 µM with an R^2^ of 0.9988. The real sample of six chewable vitamin C tablets was tested, and the relative standard deviation (RSD) was averaged at only 3.4%. It was also visualised that the IF was 3.5 using 100 µM ascorbic acid, whereas the mean cross-reactivity of several interferences, including L-histidine, uric acid, L-tryptophan, and glucose, was 17.9%.

Another PPY-based MIP has also been reported for ascorbic acid detection, which was modified on a new two-dimensional (2D)-layered, graphene-like black phosphorene quantum dots (BPQDs) on poly(3,4-ethylenedioxythiophene) nanorod (PEDOTNR) layers that were modified on GCE (PPY-BPQDs/PEDOTNRs/GCE) [35]. The imprinting was performed using CV (−0.5 V to 0.8 V, 10 cycles, 50 mV/s), using pyrrole as the monomer and ascorbic acid as the template in a 5:1 monomer–template molar ratio in 0.01 M LiClO_4_. Then, the template was removed by CV from −0.3 V to 0.5 V in phosphate buffer (pH 8.5). The detection of ascorbic acid was found to be linear from 0.01 mM to 4 mM (R^2^ = 0.9912) with a detection limit of 3.3 µM. The IF was calculated at 1.6 based on the reported sensitivity of both MIP and NIP. The mean cross-reactivity was 5.0% when ascorbic acid (0.1 mM) was tested with glucose, nicotinic acid, caffeic acid, and folic acid at 1:50 molar ratios of each interfering compound. The analysis of soft drink samples resulted in 94% to 99% recoveries.

Another successful detection of ascorbic acid was achieved using an organic electrochemical transistor (OECT) with a bare GE that was modified with electro-polymerised poly(o-phenylenediamine) (poPD) (poPD/OECT/GE) using o-phenylenediamine (oPD) as the monomer and ascorbic acid as a template with a molar ratio of 1:2 in PB (0.2 M, pH 5.2) using CV (0.0 V to 0.8 V, no cycle number mentioned, 50 mV/s) [36]. The template was removed by immersing the eMIP-modified electrode in ultrapure water. The detection of ascorbic acid was based on the current change of the OECT gate electrodes with poly(3,4-ethylenedioxythiophene) doped with poly(styrenesulfonate) (PEDOT:PSS), modified on patterned gold–nickel (Au/Ni) source and drain electrodes, where it was able to detect ascorbic acid in vitamin C beverages diluted in PB (0.1 M, pH 7.0) from 1 µM to 100 µM (no R^2^ provided) with a 10 nM detection limit. The IF was calculated at 13.5 based on the visualised results. The selectivity of the eMIP-modified electrode for ascorbic acid (10 µM) was studied in contrast with other interfering compounds and ions, including aspartic acid (100 µM), glycine (100 µM), glucose (100 µM), uric acid (100 µM), glutathione (100 µM), hydrogen peroxide (100 µM), potassium ion (K^+^, 1 mM), sodium ion (Na^+^, 1 mM), calcium ion (Ca^2+^, 1 mM), magnesium ion (Mg^2+^, 1 mM), and iron (II) ion (Fe^2+^, 1 mM), where the mean cross-reactivity was 12.6%. Several ascorbic acid concentrations were spiked into the vitamin C beverage sample, resulting in a mean RSD of 10.0%.

A poPD-based MIP was also reported for ascorbic acid detection with different modifications using GCE [37]. The electro-polymerisation was performed using oPD as a monomer on the gold nanoparticles (AuNPs)-modified MWCNTs on the GCE surface (poPD/MWCNTs/AuNPs/GCE) by CV scan (−0.5 to 1.0 V, 20 cycles, 50 mV/s). The polymerisation solution was prepared in a 5:1 oPD-ascorbic acid as a monomer–template molar ratio containing 0.1 M KCl. The template was removed by immersing the poPD/MWCNTs/AuNPs/GCE in an ethanol–water mixture at a 4:1 (*v*/*v*) ratio for 12 min. The study reported a two-order calibration for ascorbic acid detection, i.e., 0.01 µM to 2 µM (R^2^ = 0.9912) and 2 µM to 100 µM (R^2^ = 0.9980), which shows a distinct ascorbic acid peak at around 0.0 V after a DPV scan. The two calibrations produced a detection limit of 2 nM. The study also compared the signal of ascorbic acid (20 µM) between MIP and NIP, which resulted in an IF of 21. The mean cross-reactivity of the eMIP-modified GCE was only 1.7% using D-glucose, caffeine, dopamine, and uric acid. Human serum was tested using the poPD/MWCNTs/AuNPs/GCE, which showed an excellent recovery of ascorbic acid from 95.6% to 108.3%.

A very different poPD-based MIP for ascorbic acid detection method was accomplished using ratiometric electrochemiluminescence (ECL) detection, combined with MIP, based on a closed bipolar electrode (BPE) [38]. The electrode surface was electro-polymerised by CV (0.0 V to 0.8 V, 15 cycles, 50 mV/s) using oPD as a monomer and ascorbic acid as a template in PB (0.1 M, pH 5.2) with a molar ratio of 1:2, respectively. The BPE was set up using zinc indium sulphide nanoflower (ZnIn_2_S_4_) as a cathode and the MIP of poPD as an anode; both were modified on a bare graphite rod electrode. The cathode was immersed in PB (pH 7) containing 0.1 M potassium persulfate (K_2_S_2_O_8_). In contrast, the anode was immersed in PB (0.1 M, pH 7) containing tris(bipyridine)ruthenium (II) ion (Ru(bpy)_3_^2+^, 0.125 mM) and tri-n-propylamine (6.25 mM) during detection. The template removal was performed by immersing the eMIP-modified electrode with ultrapure water. The signal of ascorbic acid respective to its concentrations was studied by measuring the ECL current from the redox reaction of CV scans (0.0 V to 1.2 V, single scan, 50 mV/s) occurring on both the anode (observed wavelength, λ = 440 nm) and cathode (λ = 605 nm), simultaneously, after 2 min of incubation. The detection of ascorbic acid was calculated based on the current ratio of the cathode and the anode signal produced (I_cat_/I_ano_). In a real sample, the detection was performed on newborn calf serum with a linear detection range from 50 nM to 3 μM (R^2^ = 0.9820) and a detection limit of 20 nM, with a 0.7% mean RSD. The study also tested the effect of ascorbic acid signal versus other interfering compounds of 500 nM of urea, uric acid, dopamine, glucose, and their mixture solution with ascorbic acid, in which the mean cross-reactivity was 22.5%. However, there was no reported IF or any data to calculate the IF provided.

Another type of vitamin was also reported. Cholecalciferol was successfully detected in human plasma samples using the MIP originated from p-phenylenediamine (pPD)-resorcinol (RES) mixtures, which was modified on the screen-printed carbon electrode (SPCE) (pPD-RES/SPCE) [39]. The polymerisation solution containing 70% methanol was prepared by mixing pPD, RES, and cholecalciferol at a monomer–template molar ratio of 13.3:13.3:1, respectively, in the presence of acetate buffer (0.1 M, pH 5.2). The electro-polymerisation was performed using CV (0.0 V to 0.8 V, 7 cycles, 50 mV/s). The template was removed using acetonitrile–0.2 M hydrochloric acid (HCl) at a 10:1 (*v/v*) ratio. The linear detection of cholecalciferol was from 0.01 nM to 2 nM (R^2^ = 0.995), with a detection limit of 1 pM. The study compared the signal of MIP and NIP but only stated that NIP produced no signal. Similarly, the interference of 17-beta estradiol, dexamethasone, and betamethasone towards cholecalciferol was only described as non-interfering. However, the authors did not show any data to represent the value of IF and cross-reactivity study. The cholecalciferol detection on plasma samples exhibited an excellent recovery of 90.0% to 103.3% when spiked with 10 nM—200 nM of cholecalciferol.

The vitamin D metabolite was also studied for the detection of calcifediol in serum [28]. Initially, a GCE was modified with copper cobaltite combined with nitrogen-doped carbon nanotubes (CuCo_2_O_4_/N-CNTs) mixed with phosphorus-doped graphene oxide (P-GO). The GCE was modified by drop casting the nanocomposite on the GCE working electrode (CuCo_2_O_4_/N-CNTs/P-GO/GCE). This modified electrode was then electro-polymerised with calcifediol-imprinted PPY by 10 cycles of CV from −0.6 to 0.1 V at 10 mV/s, forming a PPY/CuCo_2_O_4_/N-CNTs/P-GO/GCE. The polymerisation solution was maintained in phosphate buffer (0.0075 M, pH 8.5) at a 1:2 template–monomer mole ratio. Subsequently, the template was removed by CV scan (0.1 V to 1.0 V, 20 cycles) in phosphate buffer (0.05 M, pH 7.0). This study highlighted the detection of calcifediol from 0.002 µM to 10 μM (R^2^ = 0.9972) with a detection limit of 0.38 nM. The serum sample analyses resulted in 80.0% to 106.4% recovery. Several compounds were tested in the selectivity study, including ergocalciferol, cholecalciferol, sodium deoxycholate, and cholesterol, and it was found that the mean cross-reactivity of the interferences was 17.0%, which may be attributed to the similarity in chemical structures of the interferences with calcifediol. However, no comparison of MIP and NIP signal was made in which the IF cannot be determined.

An eMIP-modified GCE was designed by using co-electro-polymerisation of poly(3,4-ethylenedioxythiophene) (PEDOT) with 2D-layered tungsten sulphide nanosheet (WS_2_) on a surface of single-walled carbon nanotubes (SWCNTs), with graphene oxide (GO)-modified GCE (PEDOT-WS_2_/SWCNTs-GO/GCE) for the detection of riboflavin in vitamin B_2_ tablets [40]. The electro-polymerisation was conducted using CV (0.0 V to 1.2 V, 5 cycles, 50 mV/s) with a polymerisation solution of 3,4-ethylenedioxythiophene (EDOT) mixed with riboflavin at a 2:1 molar ratio in a water solution containing 0.8 mg/mL WS_2_ and 0.04 M LiClO_4_. The template was then removed by soaking the PEDOT-WS_2_/SWCNT-GO/GCE in a 50 °C sodium hydroxide (NaOH) solution for 8 h. The calibration of riboflavin produced by LSV scan, recorded at –0.45 V of oxidation response, ranged from 0.002 µM to 0.9 µM (R^2^ = 0.9936) with a detection limit of 0.7 nM. Based on the provided calibration curve in both MIP and NIP, the IF was calculated at 3.0. In contrast, the mean cross-reactivity was found to be 4.0%, involving interfering compounds of thiamine, nicotinamide, pyridoxine, cyanocobalamin, ascorbic acid, UA, acetaminophen, and glucose, each at a 10:1 molar ratio with riboflavin. The real sample analysis recovered from 93.7% to 99.5% when spiked with riboflavin.

Another GCE-based MIP was modified using mixed monomers of catechol (CAT) and p-aminophenol (pAP) in PB (0.01 M, pH 7) containing riboflavin, dopamine, and L-tryptophan as templates with a molar ratio of 1:5 between mixed monomers and each of the templates [41]. However, the authors did not mention the template removal technique. Nevertheless, the eMIP was performed using CV (−0.8 V to 1.2 V, 10 cycles, 50 mV/s) on the bare GCE, producing a modified electrode of CAT-pAP/GCE. The modified electrode successfully detects riboflavin (visualised E vs. Ag/AgCl at −0.5 V), dopamine (visual E vs. Ag/AgCl at 0.2 V), and L-tryptophan (visual E vs. Ag/AgCl at −0.7 V) in both milk and serum samples. The calibration range was reported from 0.005 µM to 500 µM (R^2^ = 0.9966) for riboflavin, 0.05 µM to 500 µM (R^2^ = 0.9949) for dopamine, and 0.1 µM to 250 µM (R^2^ = 0.9981) for L-tryptophan, using direct detection of DPV scan (−0.7 V to 0.8 V), with detection limits of 0.0016 µM, 0.016 µM, and 0.03 µM, respectively. The mean RSD of milk and serum samples was 2.3% for riboflavin, 3.0% for dopamine, and 2.5% for L-tryptophan. However, no comparison was made with the target compound signals using MIP versus NIP. Thus, no IF can be calculated. The interference study was conducted by introducing the three target compounds with varying concentrations for each compound. It was found that the mean cross-reactivity of riboflavin, dopamine, and L-tryptophan was at 0.0% due to the specificity of each target compound at a different potential, thus giving a very minimal effect on the signals. However, an inferior cross-reactivity result, near 100% based on visual observation, was noted for all three target compounds when each of the target compounds was studied against interference from compounds such as glucose, tyrosine, uric acid, and ascorbic acid. The study also mentioned that the concentration of each of the interfering compounds was 100-fold higher than the target compounds. A similar concentration ratio must be used to reflect the true representation of the interference study.

Folic acid had also been detected using a PPY-based MIP on a GCE modified with molybdenum carbide nanoparticles (PPY/Mo_2_C/GCE) [42]. This was performed by immersing the Mo_2_C/GCE into a polymerisation solution containing folic acid as a template and pyrrole (pH 7.2) as a monomer at a molar ratio of 4:1 between monomer and template in the presence of LiClO_4_ (0.1 M). Later on, electro-polymerisation was performed using CV (−0.9 V to 1.0 V, 10 cycles, 100 mV/s). The template was then removed by immersing the eMIP-modified electrode in an ethanol and acetic acid mixture solution (90:10, *v/v*) with mild stirring. A direct electrochemical detection for folic acid using DPV (scan range parameters were not revealed) was successful in a real sample of pharmaceutical drugs and serum in 0.1 M potassium chloride (KCl) and 0.1 M PB (pH 6.5) solution, with a concentration from 0.01 µM to 120 µM (R^2^ = 0.9960) and a detection limit of 4 nM. The RSD of the real sample analysis was 3.4% for pharmaceutical drugs and 4.3% for serum. The study also reported an excellent IF of 14.6 for the detection of folic acid using the eMIP-modified electrode. The selectivity study was employed with folic acid (1 µM) against progesterone, cortisol, lactate, uric acid, ascorbic acid, dopamine, and glucose, each at 50 µM of interfering compounds. The mean cross-reactivity study showed an outstanding result of only 6.3%.

One study also reported the development of eMIP for folic acid using o-aminophenol (oAP) as a monomer on reduced graphene oxide (rGO)-modified AuNPs on GCE, denoted as poAP/rGO/AuNPs/GCE [43]. This method, with its two orders of calibration ranging from 0.02 µM to 0.8 µM (R^2^ = 0.9860) and from 0.8 µM to 10 µM (R^2^ = 0.9888), as well as a detection limit of 2.8 nM of folic acid, has successfully detected folic acid (potential, E vs. Ag/AgCl at −0.7 V) in infant formula milk, multivitamin tablets, and serum samples using direct detection of differential pulse voltammetry (DPV) scans from −1.0 V to 0.0 V, which had a mean RSD of 3.1% for the three samples. The process involves the preparation of the MIP by first modifying GCE with chemically modified rGO/AuNPs. Then, it was electro-polymerised with oAP-containing folic acid at a 1:1 molar ratio in KCl (0.1 M) using CV (−0.5 V to 1.0 V, 20 cycles, 50 mV/s). The folic acid template was removed by immersing the poAP/rGO/AuNPs/GCE in a 4:1 (*v*/*v*) ethanol–water mixture for 12 min. The IF of folic acid was found to be around 15 (based on the visualised diagram). The selectivity of the modified sensor was tested using several compounds, including pteroic acid, caffeine, theobromine, and ascorbic acid, where the mean cross-reactivity of the interferences was about 6.8%, indicating a very high selectivity of the poAP/rGO/AuNPs/GCE towards folic acid against the tested interferences.

Lastly, the imprinting of poly(3,4-ethylenedioxythiophene) (PEDOT) using 3,4-ethylenedioxythiophene (EDOT) as monomer was successful on GCE for the trace analysis detection of vitamin K in the form of vitamin K_3_, also known as menadione, in poultry drug samples [44]. The eMIP was modified using the typical three-step procedure of (1) dissolving EDOT and menadione at a molar ratio of 1.3:1, respectively, in acetonitrile containing tetrabutylammonium tetrafluoroborate (Bu_4_NBF_4_, 0.2 M); followed by (2) electro-polymerisation of PEDOT on GCE using CV (0.0 V to 1.4 V, 20 cycles, 100 mV/s) to produce PEDOT/GCE; and finally (3) template removal by immersing the eMIP-modified electrode in ethanol for 15 min while stirring. The detection of menadione was performed directly using LSV (−0.50 V to 0.0 V) from 0.009 μM to 35 μM (R^2^ = 0.9923) with a detection limit of 0.31 nM in PB (0.1 M, pH 7.0), in which the oxidation peak was noted from −0.30 V to −0.4 V at the reported concentration range. The selectivity of the PEDOT/GCE electrode was studied using several compounds against menadione (0.5 μM), including vitamin K_1_ (phylloquinone), 2,3-dichloro-1,4-naphthoquine (DINA), and 2-amino-3-chloro-1,4-naphthoquine (ACNA) at 0.5 mM, after which the mean cross-reactivity was calculated at 29.9% when observed visually. Moreover, the IF of the eMIP-modified electrode was 3.4. The poultry drug resulted in a mean RSD of 1.7% when spiked with several menadione concentration levels.

Overall, only a few vitamins were explored for the past 10 years using an in situ electro-polymerisation technique, which only includes WSV of ascorbic acid, riboflavin, and folic acid, as well as FSV of cholecalciferol, calcifediol, and menadione. Except for calcifediol, all other vitamins were electrochemically active compounds (Table 3). This means the detection of these vitamins can be implemented directly based on the signal produced by the vitamin, making the detection procedure straightforward. On the other hand, the detection of non-electrochemically active vitamins would require observation of redox indicator signals such as ferrocyanide/ferricyanide mixtures for the leached eMIP-modified electrode, and after the eMIP-modified electrode was adsorbed with the target to obtain the net reading [54,55]. Moreover, the development of detection for electrochemically active vitamins can be adapted into a portable-based detector that can be brought to the site for real-time detection. Thus, a derivatisation step is recommended for non-electrochemically active vitamins to become electrochemically active.

Typical monomers were employed for the electro-polymerisation, including CAT, EDOT, oAP, oPD, pyrrole, pAP, pPD, and RES. This was due to the nature of the listed monomers being able to undergo self-polymerisation when initiated electrochemically. The advantage of using self-polymerisation monomers is the ability of the monomer to form an interlink without the presence of a crosslinker [56,57]. During electro-polymerisation, the crosslinker would require additional experimental optimisation for the best molar ratio to be mixed with the monomer(s) and template, increasing the difficulty of the study. Thus, the utilisation of self-polymerised monomers was preferable.

The use of bare electrodes was important due to their contribution to sensitivity and the feasibility of further modification. Modifying electrodes was often performed to enhance their sensitivity by incorporating highly sensitive materials, including AuNPs, CA, CuCo_2_O_4_, GO, N-CNT, Mo_2_C, MWCNTs, P-GO, rGO, and WS_2_. Thus, the most popular electrode chosen in this review was GCE. This was due to the higher surface area of GCE compared to common graphite materials, and the cost is affordable [58,59].

However, not one study addressed the need to optimise the concentration of the polymerisation solution. This may be due to the use of a higher starting concentration of the polymerisation solution in the millimolar range (4–50 mM), which was sufficient enough to cover the surface of the working electrode during electro-polymerisation. Nevertheless, optimising the concentration of monomer in the polymerisation solution can be a good way to find the most effective adsorption of MIP on the working electrode surface, which in turn can maximise the eMIP-modified electrode detection ability. Utilising the optimisation software, such as response surface methodology (RSM), is also a viable option to save time and resources of the study [60,61].

Nonetheless, these reported studies were still able to produce a very good IF ranging from 1.6 to 21.0 and cross-reactivity of 0.0% to 29.9%. The IF value of more than 1 (IF > 1), indicating the specificity of detection towards the target compound, was good enough to differentiate between target and non-target compounds [48], whereas the low percentage of cross-reactivity indicated very selective detection for the target compound. These data further support that the use of monomers in a millimolar range was adequate.

The reported studies utilised several techniques for template removal, including immersing the eMIP-modified electrode in water, a water–solvent mixture, a pure solvent, acidic water, alkali, or a buffer solution with CV scans. The choice of method depended on the specific template and monomer used. Therefore, a thorough optimisation step is essential to enhance the accessibility of the MIP cavity, thereby improving the overall performance of the eMIP-modified electrode.

Also, there were several types of samples covered by the studies included in this review such as supplement tablets, poultry and pharmaceutical drugs, soft drinks, beverages, milk, infant formula, human and calf serum, and human plasma. The variety of samples indicates that the detection technique using in situ electro-polymerisation was applicable to any samples for vitamin detection, including food matrices and biological samples. These data provide insight into the wide possibility for the detection of vitamins in broader sample types.

In summary, Table 4 below presents a more detailed comparison of various eMIP-modified electrodes used for different types of samples to detect vitamins, along with their respective detection limits.

The most-studied vitamin was ascorbic acid. This was presumably due to its electrochemically active nature, which could be detected directly using an electrochemical sensor, as well as the simplicity of the sample preparation procedure. The development of several different eMIP-modified electrodes, however, reported higher variability in detection limits ranging from 3000.00 nM down to 2.00 nM. The lowest detection limit was represented by poPD/MWCNTs/AuNPs/GCE, which was used to detect human serum. This highly sensitive detection resulted from the utilisation of nanomaterial layers of MWCNTs and AuNPs on the bare GCE. It was reported that the use of nanomaterials greatly enhanced the detection sensitivity of the electrodes due to their high surface area and fast electron transfer [62,63,64].

Another vitamin, cholecalciferol, was detected using pPD-RES/SPCE in human plasma with a detection limit of 0.01 nM. This study reported the simplest electrode modification thanks to the use of SPCE, which offered the closest example and most promising application for portable-based detection.

Calcifediol, a metabolite of cholecalciferol, was also studied in human serum using PPY/CuCo_2_O_4_/N-CNT/P-GO/GCE, producing a detection limit of 0.38 nM. Similarly, the use of nanomaterial layers of CuCo_2_O_4_, N-CNT, and P-GO on the bare GCE contributed to excellent detection sensitivity.

For riboflavin, the lowest detection limit of 0.70 nM was achieved by PEDOT-WS_2_/SWCNTs-GO/GCE for the detection of vitamin B_2_ tablets. The use of nanomaterials, such as SWCNTs-GO, also contributed to the sensitivity.

Additionally, the incorporation of rGO and AuNPs in poAP/rGO/AuNPs/GCE for folic acid detection yielded a detection limit of 2.80 nM in various samples of infant formula, multivitamin tablets, and human serum.

Lastly, another simple modification of PEDOT/GCE was reported for the detection of menadione in a poultry drug, with a detection limit of 0.31 nM.

These findings collectively highlighted the significant influence of electrode materials and sample types on detection sensitivity. Consistently, the application of nanomaterials and hybrid composites enhanced analytical performance across the different vitamins tested.

Be that as it may, minimal reported studies also limit this review’s findings. More studies need to be conducted to discover the potential of the in situ electro-polymerisation technique, as this will help improve future technology, especially in the application of portable-based detection and POCT devices.

**Figure 3:**
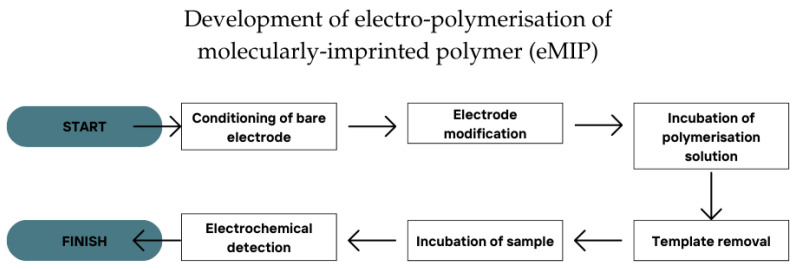
The six steps of procedure for the development of eMIP.

As a whole, the procedures of the in situ electro-polymerisation technique were divided into six general steps (Figure 3). (1) The condition of the bare electrode surface: The surface of the working electrode was typically conditioned or pre-treated either through chemical or mechanical means. The use of conventional electrodes such as GCE or GRE often involved mechanical conditioning using gentle hand sanding with alumina slurry on a slightly rough padding [65]. On the other hand, chemical or electrochemical treatment was often used by the SPE for the surface conditioning of the working electrode [66,67]. The conditioning process roughened the working electrode surface, preparing it for the following steps. (2) Next was electrode modification. The modification of the working electrode using materials with higher surface area and higher electrochemical sensitivity was typically conducted, including the use of nanomaterials [36,43] or 2D-layered materials [28,35] to enhance the electrode performance. (3) The incubation of the polymerisation solution: Depending on the method optimisation, the electrodes could be incubated in the polymerisation solution for a certain period prior to electro-polymerisation. The incubation of monomer(s) onto the working electrode surface enhanced the possibility of the polymerisation being adsorbed by the working electrode. This also simultaneously increased the chance of interaction between the template and the monomer(s) [68]. (4) The removal of the template on the eMIP-modified electrode: The template needed to be removed from the working electrode surface before it could be used for detection. The higher efficiency of template removal increased the sensing capability of the eMIP-modified electrode. There were several methods to remove the template, including leaching using a highly polar solvent such as ethanol [44], leaching using a mixture of solvent and acid such as acetonitrile–HCl mixture [39], the use of alkali leaching such as NaOH [40], the use of buffers such as PB [34], and the use of electrochemical scanning such as a CV scan [35]. The method depended on the nature of the template used in the study. (5) The incubation of samples on the eMIP-modified electrode: The samples were usually incubated for a specific duration to increase the chance of interaction between the target and the working electrode. This increased the adsorption of the target on the working electrode, making it more sensitive for detection [69]. (6) The detection of the target using an electrochemical scan: The detection of the target could be achieved directly or indirectly. Indirect detection was often used to detect a non-electrochemically active target using a redox indicator such as ferrocyanide/ferricyanide solution [32] or methylene blue [33]. However, this approach had a more prolonged procedure as the signals observed had to be taken before and after the incubation of samples to obtain the net signal.

The in situ electro-polymerisation was a simple and easy alternative for the preparation of MIP that can be directly used as a sensor for detection when combined with electrochemical techniques. However, there are many parameters that were quite hard to control during modification. One of the main challenges was to confirm the formation of the polymer on the working electrode surface. Unless the polymer of MIP was very dense, it was not easy to ascertain which part of the whole working electrode was covered by the MIP. Consequently, it was a very tedious procedure for physical characterisation, such as SEM or FTIR, to determine the presence of the MIP on the working electrode surface, as it had to be scanned at several different and random points. Additionally, the variability of occurrence of MIP formation on the working surface electrode can cause a poor reproducibility of the eMIP-modified electrodes, thus affecting the sensing capability for validation protocols. Several factors, including the conditioning of the bare electrode, the concentration of the polymerisation solution, and the electrochemical scans and parameters, controlled the occurrence of MIP on the working electrode [70,71].

Moreover, the imprinting effect might not be clearly represented by the aforementioned instruments due to poor NIP preparation (occurrence of monomer aggregation), which can cause an overestimation of IF [51].

Another challenge was the template removal procedure. The template was easy to remove when a highly polar solvent mixed with diluted acid was used [72]. This was true when using a conventional electrode. In the case of SPEs as bare electrodes, it will depend on how strongly the bare electrode printing attachment adheres to the base; otherwise, the whole printed material will peel off altogether. Moreover, the in situ electro-polymerisation can only utilise non-covalent bonding such as the hydrogen-bonding interaction between the template and the MIP [73]. Otherwise, it was not possible to remove the template using a harsher approach, such as saponification or hydrolysis [74], because it would destroy the electrode itself. This limits the applicability of the approach, although it also relies on the type of template used for detection.

The potential of converting the established eMIP-based electrochemical sensor offered a promising pathway for the POCT device. However, several challenges had to be addressed to facilitate its real-world application. One primary concern was the stability of the sensor when dealing with complex biological matrices, such as serum, plasma, or urine, or even in food matrices, where non-specific binding from the matrices could significantly affect the sensor’s analytical performance [75,76]. Furthermore, long-term use of the eMIP-modified electrode might have caused electrode fouling when it was exposed to complicated matrices over time [77,78]. Overcoming these challenges required several strategies, such as employing different electrochemical detection approaches [79,80] or adding more steps for sample preparation, which prolonged the overall detection procedures. The stability of the POCT device was also a crucial aspect of its feasibility. Therefore, future research should concentrate on overcoming the poor reproducibility by improving the developmental formulations of eMIPs that are suitable for large-scale production.

Nevertheless, in general, in situ electro-polymerisation is still a reliable approach with straightforward procedural steps that covered most of the analyte for detection, especially vitamins detection. The development involving vitamin detection in foods will provide a platform for an easy-to-use device that is portable, and a more affordable alternative to be used in the laboratory. Similarly, vitamin detections in their metabolite form in human serum or plasma can be further developed as a POCT device, widening the possibilities for reliable alternatives.

## 4. Conclusions and Future Direction

We have examined a total of 12 published articles related to the in situ electro-polymerisation technique for the detection of vitamins in ascorbic acid, riboflavin, cholecalciferol, calcifediol, and menadione, which involved the use of several common monomers including CAT, EDOT, oAP, oPD, pyrrole, pAP, pPD, and RES. Typical concentrations of these monomers range from 4 mM to 50 mM. These monomers were electro-polymerised using electrochemical CV using bare electrodes such as GRE, GCE, GE, and SPCE. These bare electrodes were often modified with AuNPs, CA, CuCo_2_O_4_, GO, N-CNT, Mo_2_C, MWCNTs, P-GO, rGO, and WS_2_ to enhance their performance, although the un-modified bare electrodes were still applicable. The most common electrochemical detection was DPV, and LSV. The reported IF was from 1.6 to 21.0 whereas the cross-reactivity was from 0.0% to 29.9%. Several types of food and biological samples were included, such as supplement tablets, poultry and pharmaceutical drugs, soft drinks, beverages, milk, infant formula, human and calf serum, and human plasma, indicating the versatility of vitamin detection using eMIP-modified electrodes across a variety of samples. However, this limited reported study urged the need of developing more detection methods using eMIP-modified electrodes for vitamins in both food and biological matrices, especially the vitamins that have not been studied yet. The development of eMIP-based electrochemical detection will enable interest in potential applications due to its enhanced specificity and selectivity, as well as its sensitivity, which eventually improves the application of portable-based detection and POCT devices, addressing existing gaps in nutritional monitoring, clinical diagnostics, and food quality control.

## 5. Limitation

This review was limited by the total output of the published articles since there were not many reported studies conducted. Also, this review was started in December 2024. Thus, any latest published articles beyond this period were not included to retain the feasibility of the report.

## 6. Materials and Methods

This review was performed based on the guidelines provided by the Preferred Reporting Items for Systematic Reviews and Meta-analysis Protocols (PRISMA-ScR). Medical subject heading (MeSH) terms “electrochemical polymerisation” or “electropolymerisation” crossed with the terms “molecularly imprinted polymer” and “vitamin A” or “vitamin D” or “vitamin E” or “vitamin K” or “fat soluble vitamin” or “vitamin B” or “vitamin C” or “water soluble vitamin” were used to search for original papers in four databases (PubMed, Scopus, and Web of Science) from 2014 to 2024. Only available full-paper publication that were reported in English were examined. Letters to the editor, books or book chapters, reviews, and conference papers, however, were not included. Duplicate articles were also eliminated.

## Figures and Tables

**Table 1:** List of WSVs and FSVs with their respective names, dietary sources, and roles in the human body. The information in the table were adapted from reported studies [1,7,8].

Vitamin	Chemical Name	Active Metabolite Form	Dietary Source	Roles
WSV				
Vitamin B_1_	Thiamine	Thiamine pyrophosphate (TPP)	Meat, cereals, grains, tubers, roots, and other protein-rich foods	Energy metabolism (carbohydrate metabolism), nerve function
Vitamin B_2_	Riboflavin	Flavin adenine dinucleotide (FAD), Flavin mononucleotide (FMN)	Dairy products, meats, poultry, broccoli	Coenzyme in redox reactions of fatty acids and the tricarboxylic acid (TCA) cycle, energy production, antioxidant enzyme function
Vitamin B_3_	Niacin, Nicotinic acid	Nicotinamide adenine dinucleotide (NAD^+^), Nicotinamide adenine dinucleotide phosphate (NADP^+^)	Poultry, fish, whole grains, dried beans, peas	Coenzyme for several dehydrogenases
Vitamin B_5_	Pantothenic acid	Coenzyme A	Peas and beans (except green beans), red meat, poultry, fish, whole-grain cereals	Fatty acid metabolism, synthesis of acetylcholine, steroid synthesis
Vitamin B_6_	Pyridoxine, Pyridoxal, Pyridoxamine	Pyridoxal phosphate (PLP)	Fish, light meat, poultry, chicken breast, pork, eggs, whole grains	Amino acid metabolism, neurotransmitter synthesis, haemoglobin production
Vitamin B_7_	Biotin	Biotin (as coenzyme)	Liver, cauliflower (*Brassica oleracea* var. Botrytis), spinach, cheese, eggs, mushrooms (*Agaricus bisporus*), chicken breast, salmon	Carboxylation reactions, fatty acid synthesis, gluconeogenesis
Vitamin B_9_	Folic acid (synthetic) Folate (natural)	5-methyltetrahydrofolate (5-MTHF)	Nuts, broccoli and other leafy green vegetables (e.g., spinach), fortified foods (e.g., cereals), beans, liver, poultry, oranges (*Citrus sinensis* L. Osbeck)	Coenzyme in single-carbon metabolism
Vitamin B_12_	Cobalamin, Cyanocobalamin	Methylcobalamin, 5-deoxyadenosylcobalamin	Meat, fish, poultry, milk, eggs	Coenzyme in the metabolism of propionate, amino acids, and single-carbon units
Vitamin C	Ascorbic acid	Ascorbic acid	Acerola (*Malpighiae marginata*), plant foods, vegetables and fruits, potatoes, bell peppers (*Capsicum annuum*), spinach and tomatoes (*Lycopersicon esculentum* L.)	Antioxidant, collagen synthesis, immune function, iron absorption
FSV				
Vitamin A	Retinol (animal source), β-carotene (plant source)	11-cis-retinal, all-trans-retinoic acid	Animal liver, orange-fleshed sweet potato (*Ipomea batatas*), green leafy vegetables, tea (*Camellia Sinensis*), carrots, corn (*Zea mays*), eggs, meat, milk, dairy products, cod and halibut	Vision (retinal), gene expression, immune function, skin health
Vitamin D_2_	Ergocalciferol	25-hydroxyergocalciferol (non-active) Calcitriol (active)	Fortified cereals, fortified plant-based milk, mushrooms	Calcium and phosphorus regulation, bone health, immune modulation
Vitamin D_3_	Cholecalciferol	25-hydroxycholecalciferol or calcifediol (non-active) Calcitriol (active)	Fish (salmon, mackerel, tuna), egg yolk, liver, sunlight exposure	
Vitamin E	α-tocopherol	α-carboxyethylhydroxychroman (α-CEHC)	Plant leaves, wheat germ oil, milk, nuts	Antioxidant, protection of cell membranes from oxidative damage
Vitamin K_1_	Phylloquinone	Hydroquinone	Originates from green leafy plants and green vegetables	Blood clotting (cofactor for clotting factors), bone metabolism
Vitamin K_2_	Menaquinone	Menaquinol	Fermented foods	Supports bone mineralisation, cardiovascular health
Vitamin K_3_	Menadione (synthetic)	Converted to menaquinone, then menaquinol	Not found naturally; used in animal feed, synthetic	

**Table 2:** Finalised included articles for this study.

Reference	Aims	Methodology	Findings
[34]	To fabricate electrochemical, molecularly imprinted sensors for the determination of ascorbic acid.	Nanofibre membranes of cellulose acetate (CA)/multi-walled carbon nanotubes (MWCNTs)/polyvinylpyrrolidone (PVP) (CA/MWCNTs/PVP) was prepared by electrospinning technique. After being transferred to a glassy carbon electrode (GCE), the nanofibre interface was further polymerised with pyrrole through an electrochemical cyclic voltammetry (CV) technique known as PPY/CA/MWCNTs/PVP/GCE. Meanwhile, the target molecule (such as ascorbic acid) was embedded into the polypyrrole (PPY) through the hydrogen bond. The effects of monomer concentration (pyrrole) and the number of scan cycles and scan rates of polymerisation were optimised.	Differential pulse voltammetry (DPV) tests indicated that the oxidation current of ascorbic acid (the selected target) was higher than that of the structural analogues, which illustrated the selective recognition of ascorbic acid by molecularly imprinted sensors. Simultaneously, the molecularly imprinted sensors had a larger oxidation current of ascorbic acid than non-imprinted sensors in the processes of rebinding. The electrochemical measurements showed that the molecularly imprinted sensors demonstrated good identification behaviour for the detection of ascorbic acid within a linear range of 10.0–1000 μM, a low detection limit down to 3 μM (S/N = 3), and a recovery rate range from 94.0 to 108.8%.
[35]	To fabricate a novel, molecularly imprinted electrochemical sensor for ascorbic acid analysis in commercial soft drink samples.	MIP nanocomposites consisting of both conducting PPY and a new, two-dimensional layered, graphene-like black phosphorene quantum dots (BPQDs), were prepared onto the surface of conducting poly(3,4-ethylenedioxythiophene) nanorods (PEDOTNRs) via electrochemical polymerisation, forming PPY-BPQDs/PEDOTNRs/GCE. The negatively charged BPQDs and template molecules in ascorbic acid were self-assembled on the surface of the positively charged PEDOTNRs. The functional monomer of pyrrole was also self-assembled with template molecules.	The prepared imprinted sensor had a linear range of 0.01–4 mM with a detection limit of 0.0033 mM and sensitivity of 4.5254 μA.mM^−1^.
[36]	To prepare an organic electrochemical transistor sensor (OECT) with an MIP-modified gate electrode for the detection of ascorbic acid.	The combination of the amplification function of an OECT and the selective specificity of MIPs afforded a highly sensitive, selective OECT sensor. CV and electrochemical impedance spectroscopy (EIS) measurements were carried out to monitor the stepwise fabrication of the modified electrodes and the adsorption capacity of the poPD/OECT/GE electrodes (where poPD refer to poly(o-phenylenediamine)). Atomic force microscopy (AFM) was employed for examining the surface morphology of the electrodes. Important detection parameters, pH, and detection temperature were optimised.	The poPD/OECT/GE sensor detects ascorbic acid at concentrations ranging from 1–100 μM, which exhibited a low detection limit of 10 nM (Signal-to-noise ratio, S/N > 3) and a sensitivity of 75.3 μA channel current change per decade under optimal conditions.
[37]	To develop a simple, rapid, and selective strategy for the detection of ascorbic acid in various samples.	The molecularly imprinted poPD film was prepared for the analysis of L-ascorbic acid on gold nanoparticles (AuNPs)-multiwalled carbon nanotubes (MWCNTs)-modified GCE (poPD/MWCNTs/AuNPs/GCE) by electro-polymerisation of o-phenylenediamine (oPD) and ascorbic acid. Experimental parameters including pH value of running buffer and scan rates were optimised. Scanning electron microscope (SEM), fourier transform infrared (FTIR) spectra, CV, and DPV were utilised for the characterisation of the imprinted polymer film.	Under the selected experimental conditions, the DPV peak currents of ascorbic acid exhibit two distinct linear responses ranging from 0.01–2 µM and 2–100 µM towards the concentrations of ascorbic acid, and the detection limit was 2 nM (S/N = 3).
[38]	To develop a novel MIP-modified, spatial-resolved “on-off” ratiometric electro-chemiluminescence (ECL)-sensing platform based on a closed bipolar electrode (BPE) for highly accurate and selective detection of ascorbic acid.	Ascorbic acid-imprinted MIP was decorated on the anode of the BPE, and tris(bipyridine)ruthenium (II) (Ru(bpy)_3_^2+^) in the anode electrolyte served as the anode emitter, while zinc indium sulphide nanoflower (ZnIn_2_S_4_), serving as the other ECL emitter, was coated on the cathode. Rebinding of ascorbic acid at the anode promoted ECL response of ZnIn_2_S_4_ (440 nm) at the cathode. Meanwhile, the ECL response at 605 nm decreased, arising from the hindered reaction of Ru(bpy)_3_^2+^ on the anode surface.	Therefore, an “on-off” BPE-ECL-sensing platform was fabricated and showed distinguished performance in repeatability and selectivity thanks to the ratio correction effect and the specific recognition from MIP. The linear range for ascorbic acid detection is from 50 nM to 3 µM, with a low detection limit of 20 nM (S/N = 3). The assay deviation of the ratio responses largely declined by about 15 and 5 times compared with the ones from the single pole in terms of repeatability and long-term stability, respectively.
[39]	To prepare an MIP-based electrochemical sensor for cholecalciferol.	Cholecalciferol-selective MIP (pPD-RES/SPCE) was synthesised by the electro-polymerisation of p-phenylenediamine (pPD)–resorcinol (RES) mixture on the screen-printed carbon electrode (SPCE) surface in the presence of cholecalciferol molecules. The electro-polymerisation of monomers created a film deposition on the electrode surface, which absolutely suppressed the reduction of ferricyanide. The removal of the cholecalciferol creates the cavities, which caused noticeably increased ferricyanide signal, which was again suppressed after the rebinding of cholecalciferol.	It was shown that the decrease in the ferricyanide peak of the MIP electrode was affected linearly by cholecalciferol concentration. This sensor shows a linear response range of 0.01–2 nM and lower detection limit of 1 pM.
[28]	To develop an accessible, fast-response, sensitive, and selective detection method for calcifediol in serum sample.	An electrochemical sensor for the detection of calcifediol was designed based on the modification of GCE by nanocomposite of copper cobaltite (CuCo_2_O_4_)/nitrogen-doped carbon nanotubes (N-CNTs) and phosphorus-doped graphene oxide (P-GO), followed by the formation of calcifediol-imprinted PPY on the electrode surface through electro-polymerisation (PPY/CuCo_2_O_4_/N-CNTs/P-GO/GCE).	The proposed sensor successfully detected calcifediol in the range of 0.002–10 μM, with a detection limit of 0.38 nM, which was much lower than deficiency concentration (20 ng/mL; 49.92 nM).
[40]	To develop a highly sensitive electrochemical sensor based on single-walled carbon nanotubes (SWCNTs) nanocomposite electro-catalyst-supported, molecularly imprinted poly(3,4-ethylenedioxythiophene) (PEDOT) film modified with two-dimensional-layered tungsten sulphide (WS_2_) nanosheet for the detection of riboflavin.	Molecularly imprinted WS_2_-PEDOT film (PEDOT-WS_2_/SWCNTs-GO/GCE) was prepared by the electrochemical co-polymerisation of functional monomer 3,4-ethylenedioxythiophene (EDOT) in the presence of template molecule riboflavin and WS_2_ nanosheet. SWCNTs nanocomposite-modified electrode was obtained by drop-coating SWCNTs dispersion containing graphene.	Under optimised conditions, the prepared imprinted sensor displayed a good linear response to riboflavin in wide concentration ranges of 0.002–0.9 μM with a low detection limit of 0.7 nM, and was successfully applied to electrochemically detect riboflavin in drug samples with good reproducibility, repeatability, and storage stability.
[41]	This research aimed to determine the exact detection of riboflavin, dopamine, and L-tryptophan through MIP based on the electro-polymerisation method.	MIP was placed on the surface of the GCE by electro-polymerisation of monomers, such as catechol (CAT) and p-aminophenol (pAP), in the presence of all three analytes. The introduced sensor (CAT-pAP/GCE) was investigated using field emission scanning electron microscopy (FESEM), atomic force microscopy (AFM), FTIR, and electrochemical methods, such as EIS, CV, and DPV.	This sensor revealed good linear ranges of 0.005–500 μM for riboflavin, 0.05–500 μM for dopamine, and 0.1–250 μM for L-tryptophan, with detection limits of 0.0016 μM, 0.016 μM, and 0.03 μM for riboflavin, dopamine, and L-tryptophan, respectively.
[42]	Herein, the study proposed a facile chemical reduction method to synthesise the molybdenum carbide (Mo_2_C) nanoparticles and its application in the electrochemical detection of folic acid through imprinting technique.	Folic acid imprinting was carried out in the presence of pyrrole monomer over Mo_2_C-modified GCE (PPY/Mo_2_C/GCE). Raman scattering, photoelectron spectroscopy, and electron microscopy techniques were employed to study the properties of Mo_2_C nanoparticles.	The proposed sensor showed the detection behaviour for a wide range of folic acid concentrations of 0.01–120 μM, with an excellent detection limit of 4 nM and good selectivity toward folic acid as compared to other co-existing species in real samples. The fabricated PPY/Mo_2_C/GCE sensors were able to be replicated with ~1.9% relative standard deviation (RSD), and their reproduced sensor offered good repeatability (RSD; 1.6%) and stability.
[43]	To develop a method for the sensitive and selective determination of folic acid in natural sources, fortified foods, and multivitamin preparations.	An electrochemical sensor was fabricated for the analysis of folic acid, which was based on electro-polymerised, molecularly imprinted poly (o-aminophenol, oAP) film and reduced graphene oxide decorated with Au nanoparticles composites (rGO-AuNPs) (poAP/rGO/AuNPs/GCE). The transmission electron microscope (TEM), CV, EIS, and DPV were utilised for the characterisation of the imprinted polymer film.	Under the optimised experimental conditions, the proposed sensor exhibited two distinct linear responses ranging from 0.02 to 0.8 µM and from 0.8 to 10 µM towards the concentrations of folic acid, and the detection limit was found to be 2.8 nM (S/N = 3). The molecularly imprinted film proposed was also found to exhibit comparatively high selectivity towards folic acid against structurally similar analogues, and the preparation of the sensor was simple and reproducible.
[44]	To develop a simple voltammetric sensor with high sensitivity and selectivity based on the imprinted PEDOT film, with imprinted sites as a recognition element for the trace analysis of menadione in poultry drug samples.	One-step electro-polymerisation of commercially available monomer EDOT in the presence of the template molecule menadione.	The imprinted PEDOT/GCE could efficiently discriminate menadione from its structural analogues, displaying good linearity with menadione concentrations in the wide range of 0.009–35 μM with a low limit of detection 0.31 nM under the optimal conditions.

**Table 3:** The summarised information for the studied articles.

Type of Vitamin	Sample	Monomer	Monomer–Template Molar Ratio	Concentration of Monomer in the Polymerisation Solution (mM)	Electro-Polymerisation Technique	eMIP-Modified Electrode	Template Removal Procedure	Electrochemical Detection	IF	Cross-Reactivity (%)	References
Ascorbic acid	Supplement	pyrrole	2.5:1	25.0	CV	PPY/CA/MWCNTs/PVP/GCE	Immersed in PB for 15 min	DPV	3.5	17.9	[34]
	Soft drink	pyrrole-BPQDs	5:1	50.0	CV	PPY-BPQDs/PEDOTNRs/GCE	Immersed in PB (pH 8.5) with CV scan (−0.5–0.8 V, 10 cycles, 50 mV/s)	DPV	1.6	5.0	[35]
	Vitamin C beverage	oPD	1:2	15.0	CV	poPD/OECT/GE	Immersed in water	Gate electrode current changed	-	12.6	[36]
	Human serum	oPD	5:1	5.0	CV	poPD/MWCNTs/AuNPs/GCE	Immersed in 4:1 (*v*/*v*) ethanol–water for 12 min	DPV	21.0	1.7	[37]
	newborn calf serum	oPD	1:2	15.0	CV	Cathode: ZnIn_2_S_4_/GRE Anode: pOPD/GRE	Immersed in water	Ratiometric ECL redox signal	-	22.5	[38]
Cholecalciferol	Huma plasma	pPD	13.3:1	4.0	CV	pPD-RES/SPCE	Template: immersed in 10:1 (*v*/*v*) acetonitrile-0.2 M HCl Target: Incubated with 20 μL of 98:2 (*v*/*v*) water–acetonitrile for three times	CV, based on indirect redox detection of ferrocyanide solution	-	0.0	[39]
Calcifediol	Human serum	pyrrole	1:2	Did not mentioned	CV	PPY/CuCo_2_O_4_/N-CNT/P-GO/GCE	Immersed in PB (0.05 M, pH 7) with CV scan (0.1–1.0 V, 20 cycles)	LSV	-	17.0	[28]
Riboflavin	Vitamin B_2_ tablet	EDOT-WS_2_	2:1	10.0	CV	PEDOT-WS_2_/SWCNTs-GO/GCE	Immersed in NaOH (50 °C) for 8 h	LSV	-	4.0	[40]
	Milk, and human serum	CAT-pAP	1:5	10.0	CV	CAT-pAP/GCE	Immersed in 1:3 (*v*/*v*) nitric acid–water	DPV	-	0.0	[41]
Folic acid	Pharmaceutical drug	pyrrole	4:1	10.0	CV	PPY/Mo_2_C/GCE	Immersed in 90:10 (*v*/*v*) ethanol–acetic acid with mild stirring	DPV	14.6	6.3	[42]
	Infant formula, mutivitamin tablet, and human serum	oAP	1:1	5.0	CV	poAP/rGO/AuNPs/GCE	Immersed in 4:1 (*v*/*v*) ethanol–water for 12 min	DPV	15.0	6.8	[43]
Menadione	Poultry drug	EDOT	1.3:1	20.0	CV	PEDOT/GCE	Immersed in ethanol with slight stirring for 30 min	LSV	3.4	29.9	[44]

**Table 4:** The tabulation of vitamin detection with their respective electrode modifications, type of samples, and detection limit.

Type of Vitamin	eMIP-Modified Electrode	Sample	Detection Limit (nM)
Ascorbic acid	PPY/CA/MWCNTs/PVP/GCE	Supplement	3000.00
	PPY-BPQDs/PEDOTNRs/GCE	Soft drink	3300.00
	poPD/OECT/GE	Vitamin C beverage	10.00
	poPD/MWCNTs/AuNPs/GCE	Human serum	2.00
	Cathode: ZnIn_2_S_4_/GRE; Anode: pOPD/GRE	Newborn calf serum	20.00
Cholecalciferol	pPD-RES/SPCE	Human plasma	0.01
Calcifediol	PPY/CuCo_2_O_4_/N-CNT/P-GO/GCE	Human serum	0.38
Riboflavin	PEDOT-WS_2_/SWCNTs-GO/GCE	Vitamin B_2_ tablet	0.70
	CAT-pAP/GCE	Milk, and human serum	1.60
Folic acid	PPY/Mo_2_C/GCE	Pharmaceutical drug	4.00
	poAP/rGO/AuNPs/GCE	Infant formula, multivitamin tablet, and human serum	2.80
Menadione	PEDOT/GCE	Poultry drug	0.31

## Data Availability

Not applicable.

## Abbreviations

5-MTHF	5-methyltetrahydrofolate
ACNA	2-amino-3-chloro-1,4-naphthoquine
AFM	atomic force microscopy
Ag	silver
AgCl	silver chloride
Au	gold
AuNP	gold nanoparticle
BPE	bipolar electrode
BPQD	black phosphorene quantum dot
CA	cellulose acetate
Ca^2^⁺	calcium ion
CAT	catechol
CuCo_2_O_4_	copper cobaltite
CV	cyclic voltammetry
DINA	2,3-dichloro-1,4-naphthoquine
DPV	differential pulse voltammetry
ECL	electro-chemiluminescence
EDOT	3,4-ethylenedioxythiophene
EIS	electrochemical impedance spectroscopy
eMIP	electro-polymerised MIP
FAD	flavin adenine dinucleotide
Fe^2^⁺	iron (II) ion
FESEM	field emission scanning electron microscopy
FMN	flavin mononucleotide
FSV	fat-soluble vitamin
FTIR	Fourier transform infrared
GCE	glassy carbon electrode
GE	gold electrode
GRE	graphite rod electrode
HCl	hydrochloric acid
HPLC	High-Performance Liquid Chromatography
IF	imprinting factor
K⁺	potassium ion
K_2_S_2_O_8_	potassium persulfate
KCl	potassium chloride
LiClO_4_	lithium perchlorate
LSV	linear sweep voltammetry
MeSH	medical subject heading
Mg^2^⁺	magnesium ion
Mo_2_C	molybdenum carbide
MS	mass spectrometry
MWCNT	multi-walled carbon nanotube
Na⁺	sodium ion
NAD^+^	nicotinamide adenine dinucleotide
NADP^+^	nicotinamide adenine dinucleotide phosphate
NaOH	sodium hydroxide
N-CNT	nitrogen-doped carbon nanotube
Ni	nickel
NIP	non-imprinted polymer
oAP	o-aminophenol
OECT	organic electrochemical transistor sensor
oPD	o-phenylenediamine
pAP	para-aminophenol
PB	phosphate buffer
PEDOT	poly(3,4-ethylenedioxythiophene)
PEDOTNR	poly(3,4-ethylenedioxythiophene) nanorod
P-GO	phosphorus-doped graphene oxide
PLP	pyridoxal phosphate
POCT	point-of-care-testing
poPD	poly(o-phenylenediamine)
pPD	p-phenylenediamine
PPY	polypyrrole
PRISMA-ScR	Systematic Reviews and Meta-analysis Protocols
PSS	poly(styrenesulfonate)
PVP	polyvinylpyrrolidone
RES	resorcinol
rGO	reduced graphene oxide
RSD	relative standard deviation
RSM	response surface methodology
Ru(bpy)_3_^2+^	tris(bipyridine)ruthenium (II) ion
SEM	scanning electron microscope
SPCE	screen-printed carbon electrode
SPE	screen-printed electrode
SWCNT	Single-walled carbon nanotube
TCA	tricarboxylic acid
TPP	thiamine pyrophosphate
UV	ultraviolet
WS_2_	tungsten sulfide
WSV	water-soluble vitamin
ZnIn_2_S_4_	zinc indium sulphide nanoflower
α-CEHC	α-carboxyethylhydroxychroman
